# USP30 protects against oxygen-glucose deprivation/reperfusion induced mitochondrial fragmentation and ubiquitination and degradation of MFN2

**DOI:** 10.18632/aging.202629

**Published:** 2021-02-19

**Authors:** Chunli Chen, Haiyun Qin, Jiayu Tang, Zhiping Hu, Jieqiong Tan, Liuwang Zeng

**Affiliations:** 1 Department of Neurology, Second Xiangya Hospital, Central South University, Changsha 410011, Hunan, China; 2 Department of Neurology, The Second People’s Hospital of Hunan Province, Changsha 410007, Hunan, China; 3 Center for Medical Genetics, School of Life Sciences, Central South University, Changsha 410078, Hunan, China; 4 Hunan Key Laboratory of Medical Genetics, Central South University, Changsha 410078, Hunan, China; 5 Hunan Key Laboratory of Animal Model for Human Diseases, Central South University, Changsha 410078, Hunan, China

**Keywords:** USP30, MFN2, mitochondria, oxygen-glucose deprivation/reperfusion (OGDR), ubiquitination

## Abstract

Cerebral ischemia-reperfusion induces mitochondrial fragmentation and dysfunction, which plays a critical role in the subsequent neuronal death and neurological impairment. Protection of mitochondria is an effective strategy to prevent neuronal damage after cerebral ischemia-reperfusion injury. USP30 is a deubiquitinating enzyme that localizes to the outer mitochondrial membrane. USP30 participates in the regulation of mitophagy and maintenance of mitochondrial morphology. In this study, the neuroprotective effect of USP30 and the underlying mechanisms were assessed in an ischemia-reperfusion injury model. SK-N-BE (2) cells were subjected to oxygen-glucose deprivation/reperfusion (OGDR) insult. Ubiquitination of mitochondrial proteins is increased during the early stage of reperfusion after oxygen-glucose deprivation (OGD), but the ubiquitination of cytoplasmic proteins exhibits no obvious changes. OGDR insult also induces rapid ubiquitination and degradation of the mitochondrial fusion protein mitofusin 2 (MFN2) in the early stage of reperfusion after OGD. Overexpression of MFN2 attenuates OGDR induced mitochondrial fragmentation. USP30 overexpression suppresses OGDR-induced ubiquitination and degradation of MFN2, and protects against mitochondrial fragmentation. Therefore, precisely targeting USP30 may provide a novel therapeutic strategy for cerebral ischemia-reperfusion related disorders.

## INTRODUCTION

Ischemic stroke is one of the leading causes of death and long-term disability in the world. Revascularization therapy via intravenous recombinant tissue plasminogen activator [1, 2] or endovascular mechanical thrombectomy [3, 4] is the mainstay and most effective strategy for acute ischemic stroke treatment. Even though the restoration of blood flow and reoxygenation is essential in preventing neuronal injury, it may give rise to ischemia-reperfusion injury and augment the neuronal damage partly through an enhanced oxidative stress and neuroinflammation [5, 6], which largely limits the efficacy of revascularization treatment. However, effective therapy for cerebral ischemia-reperfusion injury remains elusive.

Mitochondria are the powerhouses of the cell and play a pivotal role in energy metabolism, cell signaling, redox homeostasis and regulation of cellular life and death [7]. Cerebral ischemia-reperfusion injury induces mitochondrial fragmentation and dysfunction, which plays a key role in initiating the cellular apoptotic cascades and subsequent neuronal death. Maintaining the structural and functional integrity of the mitochondria is crucial in increasing neuronal survival and promoting neurological improvement. Targeting mitochondria has been proven to be a potential promising therapeutic strategy for cerebral ischemia-reperfusion injury [8, 9]. Amlodipine camsylate, a L-type calcium channel blocker, protects against oxygen-glucose deprivation (OGD) induced neural stem cell damage via maintaining mitochondrial structure and function [10]. However, more mitochondria-targeted approaches are urgently needed to improve the outcome of cerebral ischemia-reperfusion injury.

Mitofusin 2 (MFN2) is a key protein involved in mitochondrial fusion, mitophagy and interorganellar communication. It also participates in regulation of cell proliferation, apoptosis and many other diverse biological processes. Mutations in MFN2 lead to the development of neurodegeneration [11]. MFN2 mutations are associated with Charcot-Marie-Tooth disease type 2A (CMT2A). MFN2 agonists ameliorate dominant mitochondrial defects and normalize axonal mitochondrial trafficking in preclinical models of CMT2A [12]. MFN2 is a potential therapeutic target for disorders with impairment of mitochondrial dynamism and trafficking.

USP30 is a mitochondrion-localized deubiquitylase that removes ubiquitin moieties from target proteins. USP30 protects mitochondria and peroxisomes from damage [13]. USP30 regulates pexophagy and protects basal peroxisome from degradation [14]. USP30 also plays a key role in maintaining the integrity of the mitochondria. USP30 suppresses ubiquitin ligase parkin-dependent ubiquitylation of mitochondrial proteins and inhibits mitochondrial-induced cell death [15]. USP30 antagonizes mitophagy mediated by the parkin and PINK1. USP30 inhibition is beneficial for Parkinson's disease through promoting mitochondrial clearance to ensure a healthy mitochondrial network. USP30 is supposed to be a promising therapeutic target for Parkinson's disease [16–19]. However, the potential role of USP30 in cerebral ischemia-reperfusion injury remains largely unknown.

Therefore, on the basis of previous findings, the aims of this study were to determine: (1) whether oxygen-glucose deprivation/reperfusion (OGDR) induced mitochondrial proteins and MFN2 ubiquitination and degradation in SK-N-BE(2) cells; (2) whether overexpression of MFN2 and USP30 protected against OGDR induced mitochondrial fragmentation; and (3) whether USP30 regulated ubiquitination and degradation of MFN2 after OGDR insult. In this study, we confirmed that OGDR induced ubiquitination of mitochondrial proteins during the early stage of reperfusion. MFN2 was also rapidly ubiquitinated and degraded in the early stage of reperfusion. Overexpression of MFN2 and USP30 attenuated OGDR induced mitochondrial fragmentation, and USP30 overexpression inhibited OGDR induced ubiquitination and degradation of MFN2. Our results suggest that USP30 is a promising target for the development of innovative therapeutic regimens for cerebral ischemia-reperfusion injury.

## RESULTS

### Ubiquitination of mitochondrial proteins is increased during early reperfusion after OGD

**Figure 1:**
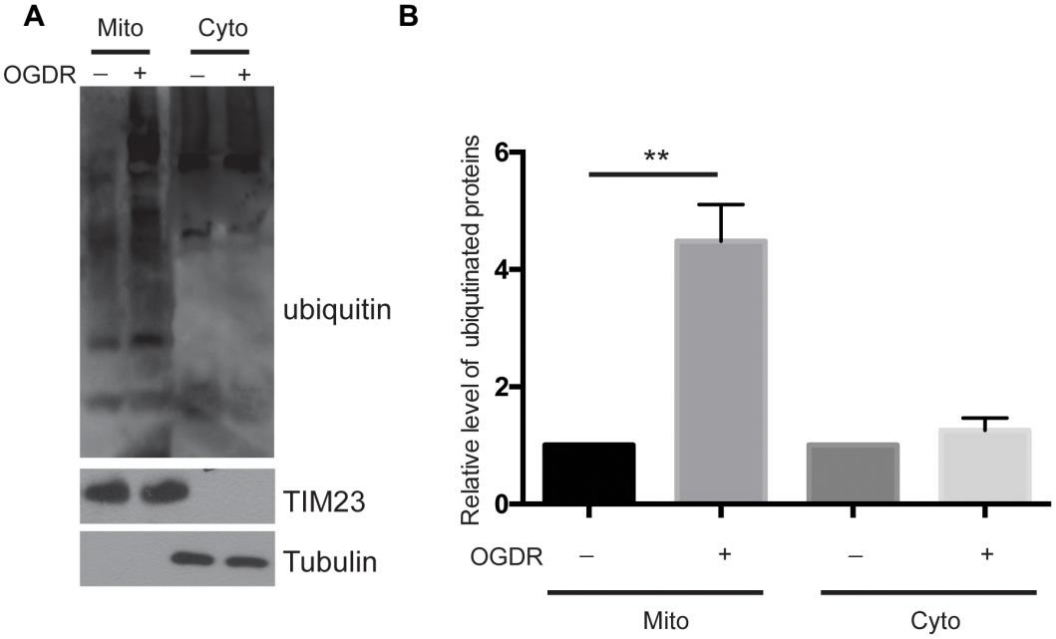
**Ubiquitination of mitochondrial and cytoplasmic proteins in the early stage of reperfusion after 4 h OGD.** (**A**) Western blot using an antibody against ubiquitin was performed to examine the ubiquitination of mitochondrial (Mito) and cytoplasmic (Cyto) proteins after 45 min reperfusion following 4 h OGD in SK-N-BE(2) cells. (**B**) Quantitation (Mean ± SEM) of A from three independent experiments. There was a significant increase in the ubiquitination of mitochondrial proteins during early reperfusion after 4 h of OGD, but not in the ubiquitination of the cytoplasmic proteins.

We used a ubiquitin antibody to explore the ubiquitination of mitochondrial and cytoplasmic proteins after OGDR insult (Figure 1). SK-N-BE(2) cells were cultured, and mitochondrial and cytoplasmic proteins were isolated after 45 min reperfusion following 4 h OGD. As demonstrated in Figure 1A, we found that the ubiquitination of mitochondrial proteins increased significantly after 45 min reperfusion following 4 h OGD(P=0.0052), while there was no significant change in the ubiquitination of cytoplasmic proteins (Figure 1A, 1B). These data suggest that mitochondrial proteins are rapidly ubiquitinated in the early stage of reperfusion after OGD.

### Ubiquitination of MFN2 is increased during early reperfusion after OGD

**Figure 2:**
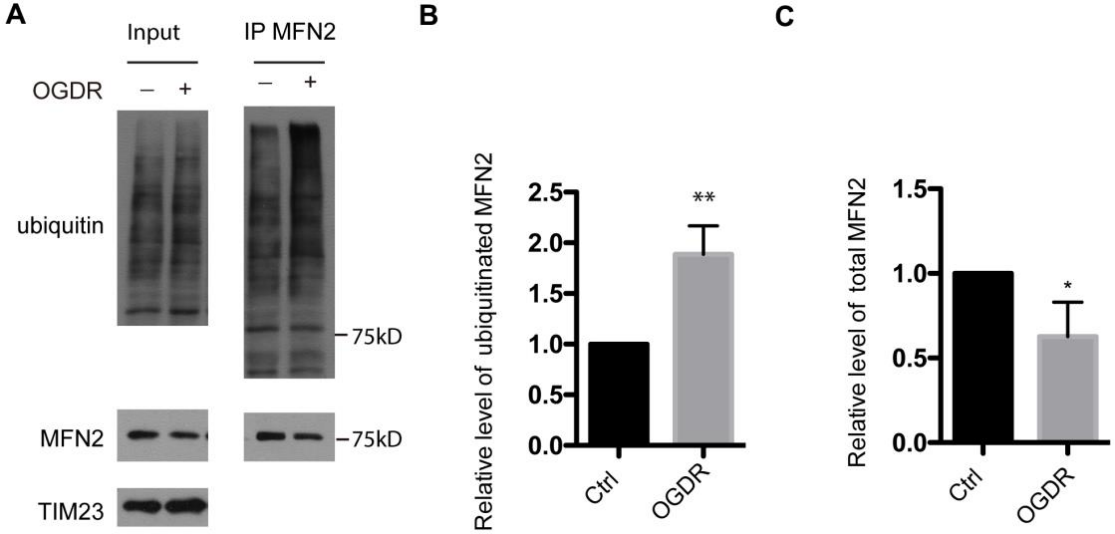
**Ubiquitination of MFN2 in the early stage of reperfusion after 4 h OGD.** (**A**) After immunoprecipitation of MFN2, western blot using an antibody against ubiquitin was performed to examine the ubiquitination of MFN2 after 45 min reperfusion following 4 h OGD in SK-N-BE(2) cells. (**B**) Quantitation (Mean ± SEM) of ubiquitinated MFN2 from three independent experiments. (**C**) Quantitation (Mean ± SEM) of total MFN2 from three independent experiments. There was a significant increase in the ubiquitination of MFN2 during early reperfusion after 4 h OGD.

MFN2 is a GTPase dynamin-like protein localized to the outer mitochondrial membrane. MFN2 participates in mitochondrial clustering and fusion. We further evaluated the ubiquitination of MFN2 after OGDR insult (Figure 2). Consistent with the above data of ubiquitination of total mitochondrial proteins, the ubiquitination of MFN2 also increased significantly after 45 min reperfusion following 4 h OGD in SK-N-BE (2) cells (Figure 2A, 2B, P=0.0034). Meanwhile, the expression of total MFN2 protein decreased significantly after 45 min reperfusion following OGD insult (Figure 2A–2C, P=0.041). This data indicates that MFN2 is rapidly ubiquitinated and degraded in the early stage of reperfusion after OGD injury.

### MFN2 is rapidly degraded during early reperfusion after OGD

**Figure 3:**
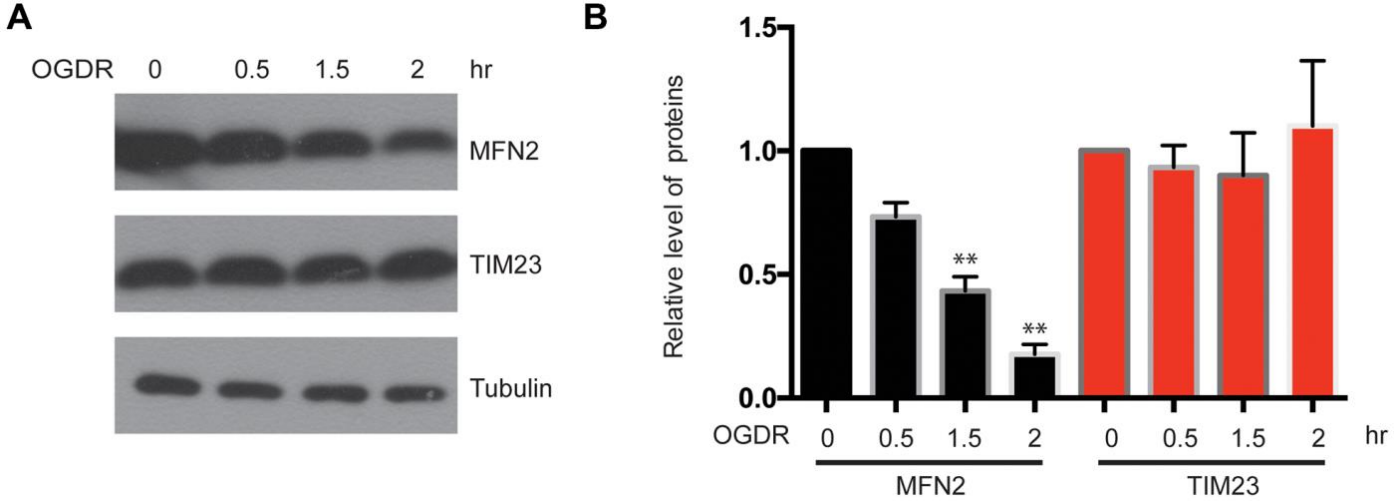
**Expression of mitochondrial outer membrane protein MFN2 and mitochondrial inner membrane protein TIM23 in the early stage of reperfusion after 4 h OGD.** (**A**) Western blot was performed to examine the protein level of MFN2 and TIM23 in the early stage of reperfusion following 4 h OGD in SK-N-BE(2) cells. Tubulin was as a loading control. (**B**) Quantitation (Mean ± SEM) of A from three independent experiments. The expression of MFN2 was reduced in a time-dependent manner during early reperfusion after OGD, whereas the expression of TIM23 was unchanged.

We have found that the ubiquitination of mitochondrial proteins and MFN2 was increased in the early stage of reperfusion after 4 h OGD, we further analyzed the expression of mitochondrial proteins after OGDR treatment. Reduced MFN2 protein expression was observed in SK-N-BE(2) cells subjected to 1.5 h (P=0.0085) and 2 h (P=0.0027) reperfusion following 4 h OGD. However, the expression of mitochondrial inner membrane protein TIM23 did not change significantly (Figure 3A, 3B). These data suggest that MFN2 is rapidly degraded in the early stage of reperfusion following OGD, which is an early event of mitochondrial damage induced by cerebral ischemia-reperfusion injury.

### Overexpression of MFN2 protects against OGDR induced mitochondrial fragmentation

**Figure 4:**
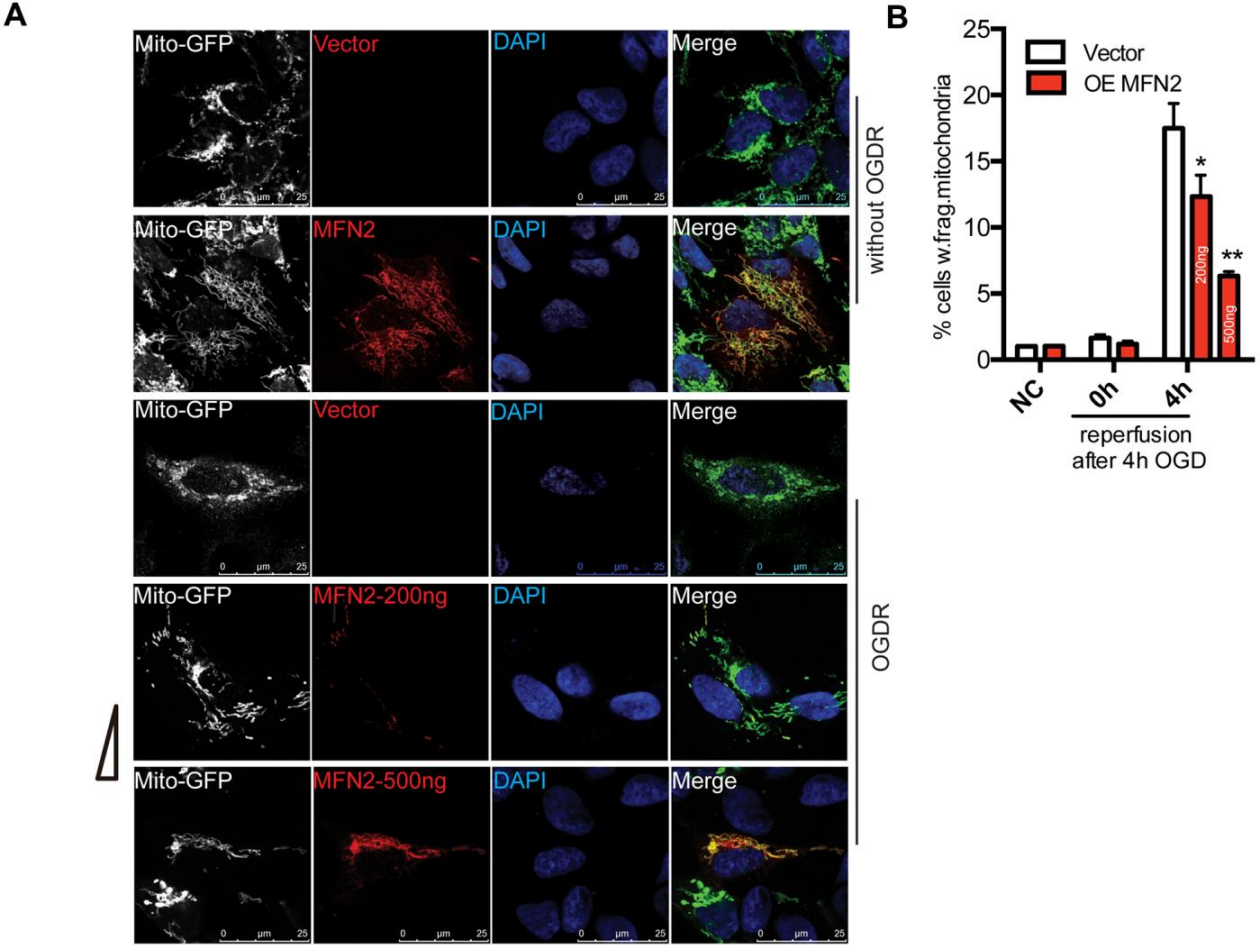
**Effect of MFN2 overexpression on mitochondrial morphology in SK-N-BE(2) cells exposed to OGDR.** SK-N-BE(2) cells were transfected with MFN2-Myc(200ng or 500ng) and Mito-GFP plasmids. After transfection 36 h, cells were treated with 4 h OGD plus reperfusion. (**A**) Digital photomicrograph under fluorescent illumination showed the morphology of mitochondria by mito-GFP. Most SK-N-BE(2) cells transfected with vector without OGDR displayed normal mitochondria, while fragmented mitochondria were evident in SK-N-BE(2) cells transfected with vector subjected to 4 h reperfusion after 4 h OGD. However, MFN2(200ng or 500ng) transfection significantly increased the number of SK-N-BE(2) cells with typical tubular and long mitochondria in a dose-dependent manner. (**B**) Quantitation (Mean ± SEM) of A from three independent experiments. Transfection with MFN2(200ng or 500ng) significantly attenuated OGDR-induced fragmentation of mitochondria in a dose-dependent manner. OE: over expression.

We overexpressed MFN2 in SK-N-BE(2) cells to determine whether intervention of mitochondrial protein degradation can play a protective role in OGDR induced mitochondrial damage. SK-N-BE(2) cells were transfected with MFN2-Myc(200ng or 500ng) and Mito-GFP plasmids. Mito-GFP was employed to indicate mitochondria. After transfection 36 h, cells were treated with OGD 4 h plus reperfusion. As demonstrated in Figure 4A, most of the cells displayed normal tubular and long mitochondria in SK-N-BE(2) cells transfected with vector without OGDR insult, while lots of mitochondria were fragmented after 4 h reperfusion following 4 h OGD in SK-N-BE(2) cells transfected with vector. However, transfection with MFN2(200ng or 500ng) significantly increased the number of SK-N-BE(2) cells with normal tubular and long mitochondria in a dose-dependent manner. MFN2(200ng or 500ng) overexpression significantly attenuated OGDR-induced mitochondrial fragmentation in SK-N-BE(2) cells in a dose-dependent manner (Figure 4A, 4B, P=0.031 and 0.0053 for 200ng and 500ng respectively). Therefore, these data strongly suggest inhibition of mitochondrial protein degradation in the early stage of reperfusion may protect mitochondria against OGDR induced fragmentation.

### USP30 overexpression ameliorates OGDR-induced mitochondrial fragmentation

**Figure 5:**
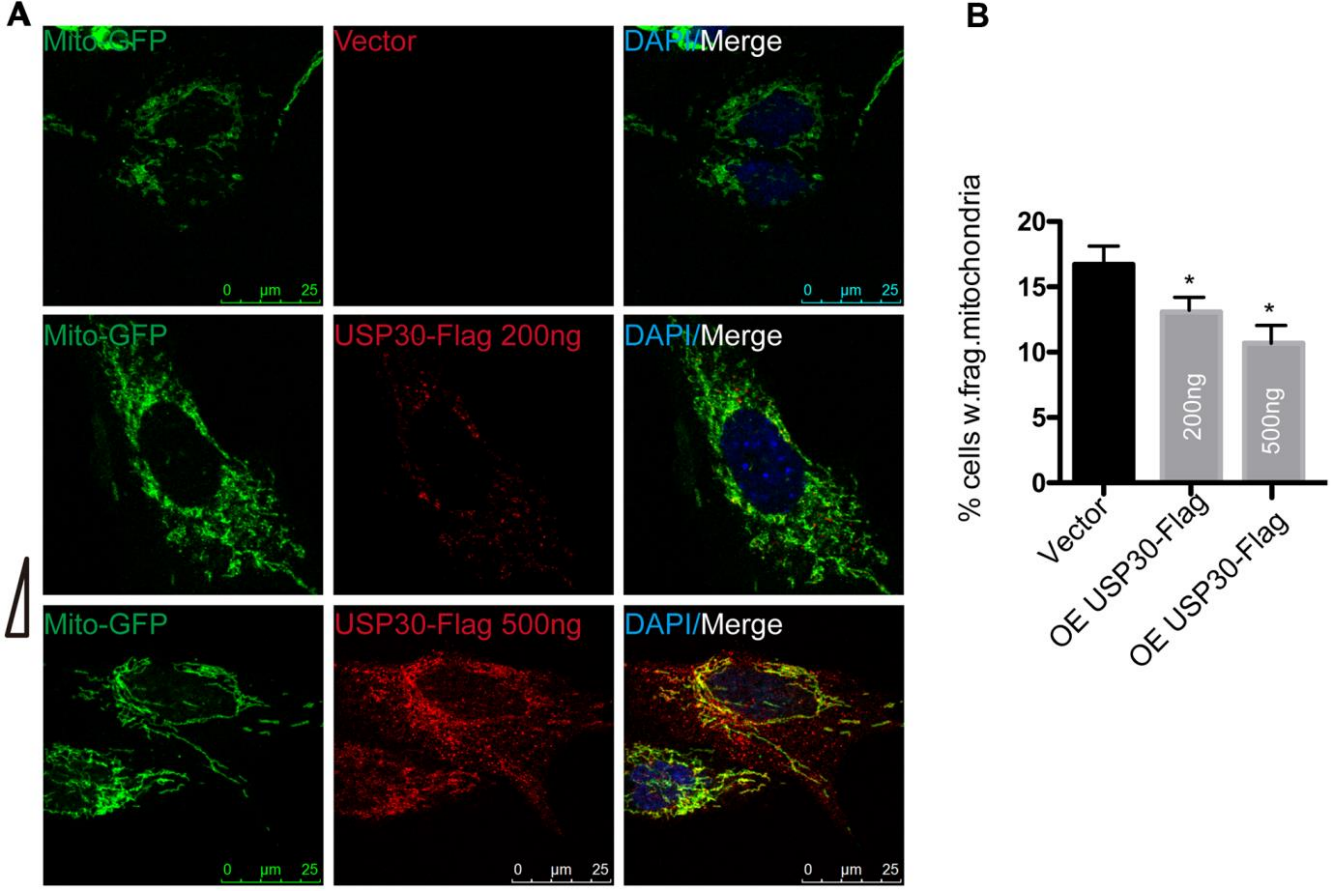
**Effect of USP30 overexpression on mitochondrial morphology in SK-N-BE(2) cells exposed to OGDR.** SK-N-BE(2) cells were transfected with indicated plasmids. After transfection 36 h, SK-N-BE(2) cells were treated with 4 h OGD plus 4 h reperfusion. (**A**) Digital photomicrograph under fluorescent illumination showed the morphology of mitochondria by mito-GFP. Mitochondrial fragmentation was evident in SK-N-BE(2) cells after 4 h reperfusion following 4 h OGD. However, USP30(200ng or 500ng) transfection significantly increased the number of SK-N-BE(2) cells with typical tubular and long mitochondria without fragmentation in a dose-dependent manner. (**B**) Quantitation (Mean ± SEM) of A from three independent experiments. Transfection with USP30(200ng or 500ng) protected against OGDR-induced mitochondrial fragmentation in a dose-dependent manner. OE: over expression.

To determine whether overexpression of USP30, a deubiquitinase localized to mitochondria, also protects against OGDR induced mitochondrial fragmentation, after transfected with USP30-Flag(200ng or 500ng) and Mito-GFP plasmids for 36 h, SK-N-BE(2) cells were subjected to OGD 4 h plus 4 h reperfusion (Figure 5). The percentage of SK-N-BE(2) cells with fragmentized mitochondria was increased after OGDR injury. However, SK-N-BE(2) cells transfected with USP30(200ng or 500ng) displayed a significant decrease in mitochondrial fragmentation after OGDR exposure in a dose-dependent manner(P=0.043 and 0.037 for 200ng and 500ng respectively). These data strongly suggest that increased USP30 expression significantly attenuates fragmentation of mitochondria after OGDR insult in a dose-dependent manner.

### USP30 overexpression inhibits ubiquitination and degradation of MFN2 after OGDR

**Figure 6:**
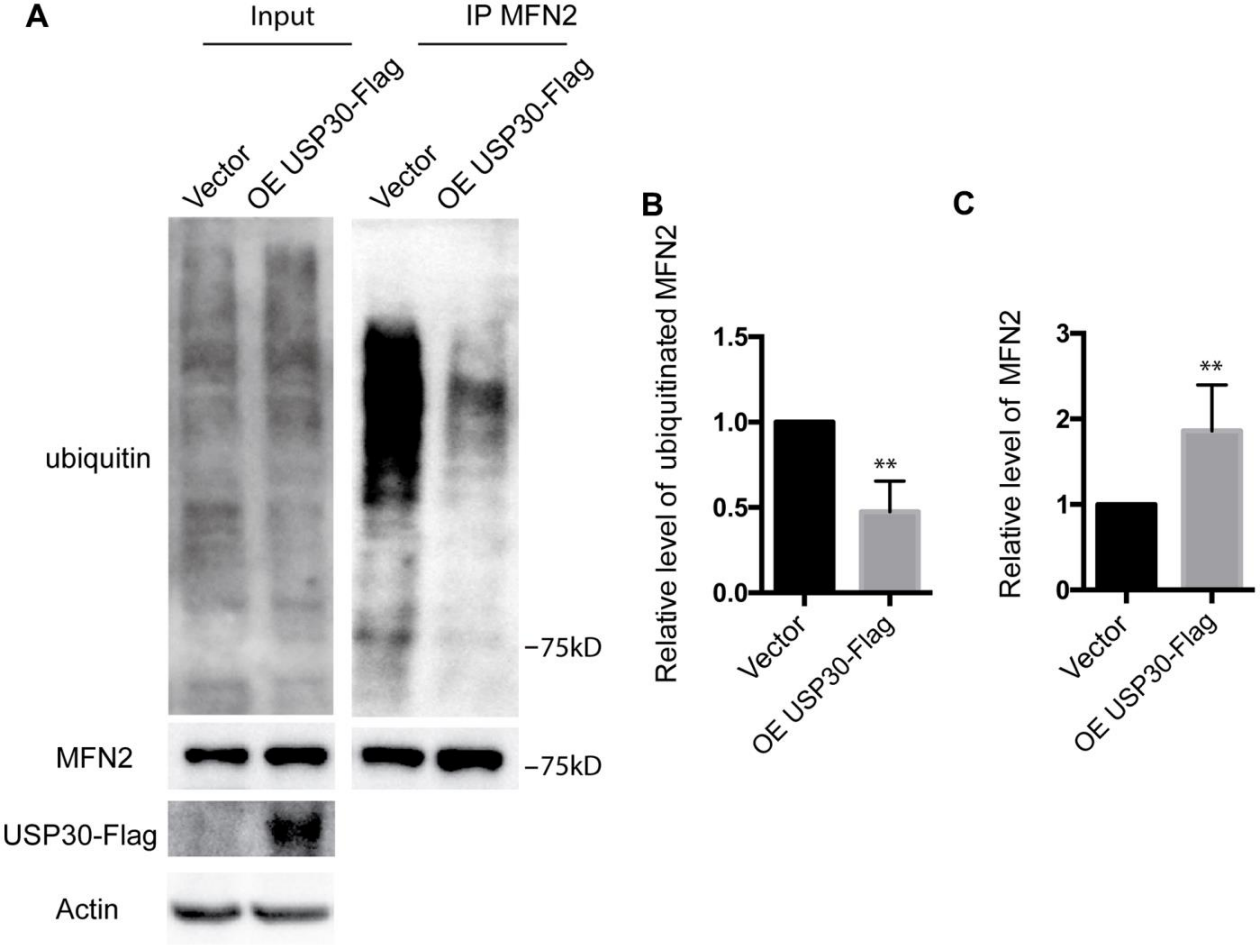
**Effect of USP30 overexpression on the ubiquitination and protein expression of MFN2 in SK-N-BE(2) cells exposed to OGDR.** SK-N-BE(2) cells were transfected with indicated plasmid. After transfection 36 h, SK-N-BE(2) cells were treated with 4 h OGD plus 4 h reperfusion. (**A**) After immunoprecipitation of MFN2, western blot was performed to examine the ubiquitination and protein level of MFN2 after 4 h reperfusion following 4 h OGD in SK-N-BE(2) cells. Transfection with USP30-Flag significantly attenuated OGDR induced ubiquitination of MFN2 and increased protein expression of MFN2. (**B**) Quantitation (Mean ± SEM) of ubiquitination of MFN2 from three independent experiments. (**C**) Quantitation (Mean ± SEM) of protein expression of MFN2 from three independent experiments. OE: over expression.

To further analyze the role of USP30 in the regulation of ubiquitination and expression of MFN2 after OGDR insult, we transfected SK-N-BE(2) cells with USP30-Flag plasmids to increase USP30 expression. After transfection 36 h, SK-N-BE(2) cells were treated with OGD 4 h plus 4 h reperfusion. As demonstrated in Figure 6, overexpression of USP30 significantly inhibited OGDR induced ubiquitination of MFN2 (Figure 6A, 6B, P=0.0029) and increased the protein expression of MFN2 (Figure 6A–6C, P=0.0085). Together with the results above, our data indicate that USP30 overexpression protects against OGDR induced mitochondrial fragmentation through regulation of the ubiquitination and expression of MFN2.

## DISCUSSION

The ubiquitin-proteasome system (UPS) and autophagy are the two complicated tightly controlled protein degradation pathways that are essential for maintaining cellular protein homeostasis. They prevent accumulation of potentially toxic proteins within the neuron and play crucial roles in neuronal survival after cerebral ischemia and many other neurological disorders [20]. Ubiquitylation serves as a degradation signal in both the UPS and autophagy systems. Cerebral ischemia-reperfusion injury induces accumulation of ubiquitin, proteasome activity impairment, and neuronal cell death [21]. Cerebral ischemia increases the expression of ubiquitin-protein conjugates in the brain. Ubiquitination level is immediately increased during reperfusion after cerebral ischemia. Elevated levels of ubiquitination is found in neurons in the cerebral ischemic penumbra with survival potential [22]. Regulation of ubiquitination may confer neuroprotection in cerebral ischemia-reperfusion injury. GS Rg1 protects against cerebral ischemia-reperfusion induced injury through suppressing proteasomal activity and ubiquitinated protein aggregation in brain [23]. The ubiquitin E3 ligase TRAF6 promotes cerebral ischemia-reperfusion injury through increased ubiquitination and activation of Rac1 [24]. However, despite recent advances, the exact role of the ubiquitylation in cerebral ischemia-reperfusion injury and the factors contributing to ubiquitinated protein aggregation still need further exploration [25]. In the study, we found that the mitochondrial proteins are immediately ubiquitinated during reperfusion after OGD. Ubiquitination of mitochondrial proteins increased as early as 45 min reperfusion following 4 h OGD. Ubiquitylation plays a key role in the maintenance of mitochondrial integrity. Therefore, increased ubiquitination of mitochondrial proteins after OGDR would lead to impairment of mitochondrial structure via continuous ubiquitination and degradation.

Cerebral ischemia-reperfusion injury decreases the protein expression of MFN2 in mice [26]. Mitochondrial release and decreased expression of MFN2 after cerebral ischemia-reperfusion injury was found in mitochondria of the cerebral cortex, but not in hippocampal mitochondria [27]. Consistent with previous studies, we found that MFN2 was immediately ubiquitinated and degraded in the early stage of reperfusion after 4h OGD. Therefore, the increased ubiquitination and degradation of MFN2 may represent an important mechanism of mitochondrial damage and neuronal death after OGDR injury.

Mitochondria play a pivotal role in neuronal survival and death via their functions in ATP production, apoptosis, reactive oxygen species generation and Ca^2+^ homeostasis. Cerebral ischemia-reperfusion induces mitochondrial fragmentation and dysfunction, which will aggravate neuronal death and is supposed to be the key pathogenic mechanism. Mitochondria play diverse roles in cerebral ischemia and are promising targets for the clinical treatment of cerebral ischemia-reperfusion injury [28]. However, the mechanisms underlying mitochondria in ischemic neuronal death and protection still need further exploration. In our study, we found that overexpression of MFN2 protected against OGDR induced mitochondrial fragmentation. MFN2 is a potential target for CMT2A and MFN2 agonists attenuate MFN2 mutations induced mitochondrial defects in CMT2A [12]. Nuclear receptor subfamily 4 group A member 1 (NR4A1) inhibition protects against cerebral ischemia-reperfusion induced mitochondrial damage through upregulation expression of MFN2 and reversing MFN2-mediated mitophagy [29]. HDAC2 knockout enhances neuronal expression of MFN2 and promotes neuronal survival in mice model of cerebral ischemia-reperfusion injury [30]. Thus, targeting MFN2 may open avenues for discovering innovative therapeutic regimens for cerebral ischemia-reperfusion injury.

USP30, a deubiquitinase localized to the mitochondrial outer membrane, is essential in maintaining the integrity of mitochondria. USP30 depletion leads to elongated and interconnected mitochondria, which is rescued by ectopic expression of USP30 [31]. USP30 removes Lys 6- and Lys 11-linked multimers from intact ubiquitylated mitochondria and regulates atypical ubiquitin chains on mitochondria [32]. USP30 inhibition confers neuroprotection in Parkinson's disease through rescuing the defective parkin-mediated mitophagy, promoting mitochondrial degradation and maintenance the integrity of mitochondria [16]. USP30 also play a key role in the regulation of BAX/BAK-dependent apoptosis and USP30 inhibition promotes mitochondrial cell death [15]. However, the potential role of USP30 in cerebral ischemia-reperfusion injury is still largely unknown. Therefore, in this study, we investigated the role of USP30 in SK-N-BE(2) cells exposed to OGDR. We demonstrated that overexpression of USP30 attenuated OGDR-induced mitochondrial fragmentation, suggesting that USP30 overexpression is beneficial for cerebral ischemia-reperfusion injury through maintaining mitochondrial structure integrity.

The ubiquitin-proteasome system plays a pivotal role in the maintenance of mitochondrial homeostasis through regulating the ubiquitination of MFN2. MITOL, a mitochondrial ubiquitin ligase, regulates mitochondrial dynamics by activating MFN2 via K192 ubiquitination [33]. Parkin, an E3 ubiquitin ligase, induces a rapid ubiquitination and degradation of MFN2 in a proteasome- and p97-dependent manner [34, 35]. Therefore, we further studied the neuroprotective mechanisms of USP30 in cerebral ischemia-reperfusion injury. We found that USP30 overexpression inhibited OGDR induced MFN2 ubiquitination and degradation. MFN2 is supposed to be a potential target for USP30. USP30 is essential for the maintenance of mitochondrial homeostasis through regulating the deubiquitination of ubiquitylated MFN2 and promoting mitochondrial fusion [36]. Therefore, the neuroprotective effect of USP30 against OGDR induced mitochondria damage may be relate to its inhibition of ubiquitination and degradation of MFN2.

In conclusion, our study demonstrates that in the early stage of reperfusion after OGD, the mitochondrial proteins are immediately ubiquitinated. The ubiquitination and degradation of MFN2 is also increased during early reperfusion after OGD, while overexpression of MFN2 ameliorates OGDR induced mitochondrial fragmentation. USP30 overexpression inhibits OGDR-induced ubiquitination and degradation of MFN2, and protects against mitochondrial fragmentation. Our results suggest that overexpression of USP30 may be a promising strategy for the treatment of disorders whose etiology is based upon cerebral ischemia-reperfusion injury.

## MATERIALS AND METHODS

### Antibodies, cells and reagents

SK-N-BE(2) cell was obtained from ATCC. Ubiquitin (#3933, #3936), MFN2(#11925), Tom20(#42406), β-tubulin and β-actin antibodies were purchased from Cell Signaling Tech. TIM23 (sc-514463) antibody was purchased from Santa Cruz Biotechnology. USP30-Flag (Addgene, #22578) and Mito-GFP (Addgene, #44385) were obtained from Addgene.

### Cell culture and transfection

SK-N-BE(2) cell line was cultured in Eagle's Minimum Essential Medium and F12 Medium (Invitrogen Life Technologies) supplemented with 10% fetal bovine serum(Sigma-Aldrich), 50 μg/mL penicillin, and streptomycin in a 5% CO_2_ incubator. Cells were transiently transfected using Lipofectamine 2000 reagent (Invitrogen Life Technologies) with the indicated plasmids MFN2-Myc(200ng or 500ng per well for 24-well plate), USP30-Flag(200ng or 500ng per well for 24-well plate) or Mito-GFP following the protocol.

### OGDR

SK-N-BE(2) cells were transferred into a temperature controlled (37° C) anaerobic chamber (Forma Scientific) containing a gas mixture composed of 5% CO_2_, 95% N_2_. The culture medium was replaced with deoxygenated glucose-free Hanks' Balanced Salt Solution (Invitrogen) and cells were maintained in the chamber for 4 h. After OGD, SK-N-BE(2) cells were maintained in culture medium supplemented with 10% FBS under normoxic culture conditions for different times.

### Mitochondrial fractionation

Cells were washed twice with ice-cold PBS, and then scraped into ice-cold PBS followed by centrifugation at 1,000 g for 5 min at 4° C. Cell pellets were resuspended in mitochondrial isolation buffer (5 mM Hepes pH 7.4, 3 mM MgCl_2_, 1 mM EGTA, and 250 mM sucrose) containing protease and phosphatase inhibitors. Lysates were passed through a 5/8-inch 25-gauge needle 20 times using a 1-mL syringe and centrifuged at 1,000 g, 4° C for 20 min. Supernatants were collected, and cytosolic extracts were recovered by centrifugation at 10,000 g, 4° C for 15 min to obtain crude mitochondrial pellets.

### Immunoprecipitation

For immunoprecipitation, cell lysis buffer included 1% Triton, 10 mM HEPES, pH7.5, 142.5 mM KCl, 5 mM MgCl_2_,1 mM EDTA, 10% glycerol, and a protease inhibitor cocktail. MFN2 antibody and Protein G agarose beads (Sigma-Aldrich) were used for immunoprecipitation. Total protein was analysed by SDS-PAGE, and a rabbit anti-ubiquitin antibody (Sigma-Aldrich) was used for immunoblotting.

### Immunoblotting

Cells were lysed with lysis buffer containing 1% Triton X-100. Samples were subsequently separated by SDS–PAGE and transferred to Immobilon-P polyvinylidene difluoride membranes (Millipore). Immunoblot analysis was performed and visualized with Super-Signal West Pico Chemiluminescent substrate (Pierce) or Immobilon Western Chemiluminescent HRP substrate (Millipore). Signal intensities were analyzed using a LAS-3000 mini imaging analyzer and Multi Gauge software, version 3.0 (Fujifilm).

### Immunofluorescence

Cells grown on coverslips were washed with PBS and fixed in 4% paraformaldehyde in PBS for 10 min at 4° C. Fixed cells were permeabilized with 0.1% Triton X-100 in PBS for 5 min, blocked with 3% bovine serum albumin in PBS for 30 min, and incubated with primary antibodies for 1 h. After washing, cells were incubated with secondary antibodies for 30 min. Images were acquired on a confocal laser microscope (FV1000D IX81, Olympus).

### Quantitative and statistical analysis

Mitochondrial fragmentation was assessed as described before [19]. Cells with shortened, punctate, and sometimes rounded mitochondria were classified as fragmented. Quantification was performed using more than 300 cells per experiment.

The data are presented as mean ± Standard Error (SEM). Paired Student’s t-test was used for comparison between pre- and post-treatment values in each group, while unpaired t-test was used for comparison between two groups. One-way ANOVA followed by Tukey multiple comparisons test was used to compare values among three or more groups. P < 0.05 was considered statistically significant. All statistical analyses were performed using GraphPad Prism (La Jolla, CA, USA).
